# Professional attractiveness among long-term care workers in nursing homes in China: a cross-sectional study

**DOI:** 10.1186/s12913-024-11023-x

**Published:** 2024-04-29

**Authors:** Xiaojing Qi, Ziyan Dong, Wen Xie, Liuqing Yang, Jie Li

**Affiliations:** 1 Department of Nursing, Peking Union Medical College Hospital, No.1 Shuaifuyuan Wangfujing Dongcheng District, Beijing, 100730 China; 2 School of Nursing, Tongji Medical College, Huazhong University of Science and Technology, Wuhan, China

**Keywords:** Long-term care, Nursing home, Staff, Professional attractiveness

## Abstract

**Background:**

The population aging trend and the shortage of elderly care workers require the long-term care profession to become more attractive. However, the professional attractiveness among long-term care workers has yet to be extensively studied. This study aims to identify the factors that influence the attractiveness of the long-term care profession for nursing home (NH) care workers..

**Methods:**

A cross-sectional study was conducted in more than 50 NHs. Perception of professional attractiveness among long-term care workers and potential associated factors were measured using the Attractive Work Questionnaire (AWQ) and structural instruments including the Fraboni Scale of Ageism (FSA) and the Maslach Burnout Inventory (MBI). A multiple linear regression method was employed to explore the influence of potential independent variables on professional attractiveness.

**Results:**

The overall response rate was 99%. The results showed the score of professional attractiveness (185.37 ± 20.034), as well as the scores of each component (99.26 ± 11.258 for work condition, 30.13 ± 3.583 for work content, and 55.99 ± 7.074 for job satisfaction). Findings of multiple linear regression analysis indicated that age(*β = 0.129, p<.05*), years of work(*β = 0.156, p<.05*), 12-hour shifts(*β = 0.185, p<.05*), and training times per year(*β = 0.148, p<.05*) positively associated with long-term care workers perceived professional attractiveness. Whereas only ageism(*β=-0.267, p<.05*) significantly and negatively influenced professional attractiveness.

**Conclusions:**

The perceived professional attractiveness of long-term care workers in NHs was acceptable. Age, years of work, shifts, training opportunities, and ageism contributed to the professional attractiveness of nursing home care workers in China. Target intervention measures should be taken to enhance the attractiveness of long-term care careers so as to avoid the shortage of long-term care workers.

## Background

Population aging has become an inevitable trend that could pose serious health challenges worldwide. The proportion of older persons in the world is anticipated to increase from the current level of 9% to nearly 16% by 2050, with 1.6 billion older people [1]. According to the National Bureau of Statistics of China data [2], the number of elderly adults aged 60 years and over reached 264 million in 2020, accounting for 18.7% of the total Chinese population. Elderly people experience a decline in cognition and function as they age, threatening independence. An estimated 47 million disabled or semi-disabled older adults live in China in 2020 [3]. Hence, there is an urgent need to develop long-term care (LTC) to address the challenges of aging and rising disabled populations.

LTC refers to care provided by carers or care workers for individuals with declines in intrinsic capacity or functional ability, which can last for an extended period [4]. LTC services generally occur in non-institutional settings such as homes and communities or institutional settings such as LTC facilities (i.e., nursing homes (NHs)). China’s long-term care service system consists of three main types of services: home-based, community-based, and institution-based, and shows a pattern of “9073”, namely, 90% of the elderly ageing at home, 7% receiving community-based services and 3% receiving institutionalized services [5]. Home care, with family members as the primary caregivers, has long been the traditional mode of care in China [6]. However, decreased fertility rates, shrinking family sizes, and frequent internal immigration from rural to urban have shifted China’s traditional family-based care model for the elderly to the current trend toward greater reliance on NHs [7].

The development of NHs faces several challenges, including low staffing ratios and high staff turnover combined with low salaries and unfavorable social attitudes toward LTC workers [8]. The National Occupational Standard for Elderly Caregivers (2011), formulated by China’s Ministry of Labor and Social Security, stipulates that the occupation of elderly care workers has four grades: junior, intermediate, senior and technician. Subsequently, in the 2019 revision, the entry requirements for elderly care workers have been further relaxed to meet the challenge of a lack of elderly caregivers, and the number of years of experience required for the promotion of care worker professional qualifications has been shortened. The new standard adjusts the educational requirement for care workers from “graduation from junior high school” to “no educational requirement”; and adjusts the declaration requirement for junior workers is adjusted from “more than 2 years of continuous apprenticeship in this occupation” to “more than 1 year (inclusive) of cumulative work in this occupation or related occupations”. For caregivers who have already obtained the intermediate elderly care worker vocational qualification certificate and want to obtain the senior vocational qualification, the number of years of employment required has been reduced from 4 to 2 years [9]. Most long-term care workers in NHs are middle-aged women with low education levels and hardly accept formal pre-employment training [10–12]. Moreover, it is frustrating that students and skilled nurses show a somewhat sluggish interest in devoting themselves to LTC [13, 14]. Previous studies examining the quality of LTC facilities have shown that many problems are linked to caregiver shortages, such as falls, pressure ulcers, and medication errors [15]. The shortage of skilled care workers reduces a unit’s ability to meet the diverse nursing requirements of the aged for physical, psychological, and rehabilitation.

The definition of professional attractiveness varies across different occupations and lacks consistency in the literature. Ateg defines “attractive work” as work that possesses positive characteristics and attracts the attention of job seekers and current employees in a positive manner [16]. Professional attractiveness of long-term care workers had previously been described based on the Walker and Avant’s classical concept analysis method [17]. The definition is that LTC facilities possess the ability to attract excellent staff, not only in terms of eliciting the willingness of potential applicants to work but in terms of retaining and motivating the current long-term care workers. According to our interpretation, professional attractiveness also reflects a psychological tendency of employees working in LTC facilities, which was driven by the work experiences and perceptions (e.g. satisfaction, turnover intention, and burnout) that various job features bring to employees.

The experience of work tends to be affected on many levels. Previous studies have demonstrated numerous individual characteristics, such as age, health status, working years, and education levels, could influence long-term care workers’ job satisfaction and turnover intention [18, 19]. Staff participation in supportive management had a positive relationship with their job satisfaction in LTC facilities [20], which might endow care workers with more confidence and sense of accomplishment. Besides, several organizational factors have been reported that might exert an influence on job satisfaction and turnover intention. Foa found that positive relationships with colleagues and training favor job satisfaction, while workload, lack of training, and reduction of rest time create dissatisfaction and even increase burnout [21]. Lee et al. reported that several work conditional factors like wage, working hours, and working intensity influence the turnover intention of home care workers [22]. Additionally, intrapersonal factors, including ageism, burnout, and mood, have been associated with job satisfaction and intention to leave directly or indirectly. Ageism, referring to the prejudice of one age group against another [23], is associated with LTC workers’ intention to leave and plays a mediating role in job satisfaction and intention to stay [24]. Actually, discrimination against the aged based on age, resources, and contribution leads to a negative image of geriatric nursing [25]. White found that those nurses who work in LTC facilities with higher burnout were more likely to leave their jobs [26]. It appears that burnout would make LTC less attractive. Long-term care workers who are dissatisfied with their current job situation or even have a desire to leave may perceive that their job has become less desirable.

There have been some studies exploring job attractiveness in the healthcare profession [27, 28], indicating that determinants of professional attractiveness are work engagement and age. However, few studies probed into professional attractiveness in long-term care workers directly. This study aimed to investigate the status of professional attractiveness among long-term care workers working in NHs in China using structural questionnaires and explore the impact of potential factors on it. We hypothesized that demographics, job security factors (including wage and training), and individual subjective factors (including job burnout and ageism) would affect long-term care workers’ professional attractiveness.

## Methods

### Study design and participants

This was a cross-sectional descriptive study conducted from June 2021 to June 2022. The sample size was grossly calculated based on the requirement of a multiple linear regression model, in which cases should be more than 5–10 times the number of independent variables [29]. The number of independent variables in this study was 15 and considering 10% invalid questionnaires, the required sample size should be 83–165 at least. The inclusion criteria for the current study were as follows: (1) care workers who work in NHs and take care of the elderly directly, (2) have worked in LTC facilities for at least three months, and (3) are volunteered to participate in this survey. The participants were excluded if they were: (1) unable to complete the questionnaire or (2) reluctant to participate in the study.

### Data collection

The study was approved by the ethics committee of Huazhong University of Science and Technology. Subjects were selected using a convenience sampling strategy from 57 NHs in Wuhan, Hubei Province, and Kaifeng, Henan Province, China. All participants were informed about the content and purpose of the study in detail and signed an informed consent form. Structural questionnaires were delivered to participants on-site by trained investigators, and confidentiality was emphasized. We recruited 396 care workers and the responsiveness of the sample was 99%.

## Variables and measurements

### Independent variables

#### Demographics and job institutional security factors

Long-term care workers’ basic demographics and job security data were obtained using a questionnaire designed by researchers, which included age, marital status, gender, education, employment form, working years, certificate level, shift mode, number of care objects, number of care objects with a disability, and whether to participate in management. Income and training opportunities belong to job security.

#### Ageism

The Fraboni scale of ageism (FSA) was used to measure cognitive and affective aspects of ageism [30]. The FSA consists of 29 items related to three dimensions of counterstatement, avoidance, and denial. Each item is evaluated on a Likert scale from 1 (definitely do not agree) to 4 (definitely agree), with higher scores suggesting greater ageism. The Chinese version of the FSA was revised previously and confirmed as a valid and reliable tool among medical students with a Cronbach’s alpha of 0.81 and a content validity of 0.93 [31]. To ensure accurate results, we have verified the reliability of FSA in long-term care workers with a Cronbach’s alpha of 0.856 and a KMO value of 0.786.

#### Job burnout

The Maslach Burnout Inventory (MBI) is one of the best-known tools for evaluating job burnout, which consists of three dimensions of emotional exhaustion, depersonalization, and personal accomplishment [32]. The Chinese version of MBI adapted by Li et al. [33] has been used widely and was constitutive of 15 items on a 7-point Likert scale ranging from 1 (never) to 7 (every day). The overall reliability of MBI in long-term care workers is acceptable with a Cronbach’s alpha of 0.882.

#### Professional attractiveness

The Attractive Work Questionnaire (AWQ), created by Ateg and Hedlund [34], was used to assess employees perceived professional attractiveness. For the purpose of the study, the Chinese version was used to assess the professional attractiveness of long-term care workers in the Chinese context after cross-cultural adjustment. The revised AWQ has 46 items, including three areas of work conditions, work contents, and job satisfaction. All items were rated on a 5-point Likert scale ranging from *not at all* to *entirely*, with higher scores indicating that the job is more attractive to the employee. The Cronbach’s alpha of the three components of the Chinese version of AWQ ranges from 0.816 to 0.920, indicating good reliability. As well as the KMO values of the three components are 0.822, 0.685, and 0.848 respectively, indicating good validity.

### Statistical analysis

Standard descriptive statistics were used to describe participants’ characteristics. Mean values and standard deviation (SD) (for symmetric distribution) or median and quartiles (for skewed distribution) were calculated for continuous variables, while frequency and percentage were used for categorical variables. We generated the mean total score for professional attractiveness and the mean score for each domain. A series of Spearman’s correlation coefficients, the Wilcoxon rank-sum test, and the Kruskal-Wallis H test were calculated to examine the association between demographics, job-related characteristics, ageism, burnout, and professional attractiveness. When the p-value was less than 0.05, it was considered to be statistically significant. The multiple linear regression method was employed to analyze the influence of many variables that were significant in the univariable analysis of professional attractiveness. All of the data were analyzed by the SPSS, version 24.0.

## Results

### Characteristics of sample

Table 1 showed the summary characteristics of the long-term care workers. The participants’ age ranged between 18 and 74 years. We found that most workers were middle-aged and had been working in LTC services for 3–7 years according to analysis. The overwhelming majority of the long-term care workers included in the analysis were female (89.3%), married (87.8%), and with a degree below associate (73%). The proportion of contract (49%) and temporary (51%) workers was comparable. Merely about one-fifth of the care workers in the sample had intermediate or advanced professional qualifications for elderly care workers. About half of the workers (47.4%) work eight-hour shifts and only around one-third of the staff are involved in management. Results indicated that the typical ageism scores ranged from 51 to 63 on a total score of 129, and the typical job burnout scores ranged from 45 to 55 on a total score of 105 in participants.

**Table 1:** Characteristics of the sample (*N* = 392)

Variables	*N* (%)/M (IQR)	Range
Age	52(46, 56)	18–74
Marital status		
Single	14(3.6%)	
Married	344(87.8%)	
Divorced	3(1.0%)	
Widowed	31(8.0%)	
Gender		
Female	350(89.3%)	
Male	42(10.7%)	
Level of education		
Primary school and below	78(19.9%)	
Junior high school	157(40.1%)	
High school/junior college	90(23.0%)	
Associate	46(11.7%)	
Bachelor’s degree and above	21(5.4%)	
Type of employment		
Contract worker	192(49.0%)	
Temporary worker	200(51.0%)	
Years of work	5(3, 7)	1–24
Certificate level of elderly care worker		
Certificateless	125(31.9%)	
Junior elderly care worker	177(45.2%)	
Intermediate elderly care worker	63(16.1%)	
Senior elderly care worker	17(4.3%)	
Technician elderly care worker	10(2.6%)	
Shift system		
8-hour	95(24.2%)	
12-hour	186(47.4%)	
24-hour	111(28.3%)	
Average number of persons cared for per day	6(5, 10)	0–35
Average number of persons with disabilities cared for daily	3(2, 5)	0–30
Whether to participate in management		
Yes	118(30.1%)	
No	274(69.9%)	
Monthly income (yuan)	3650(3025, 4000)	1200–20,000
Training times	5(3, 12)	0–24
Ageism ^a^	58(51, 63)	
Job burnout ^b^	50(45, 55)	

^a^ FSA has possible ranges of 29 to 116

^b^ MBI has possible ranges of 15 to 105

### The professional attractiveness of long-term care workers

Table 2 showed the results of the analysis for the AWQ questionnaire regarding the three aspects of professional attractiveness: work conditions, work contents, and job satisfaction. The total mean score for the AWQ was 185.37 ± 20.034 points, among which the score of work condition, work content, and job satisfaction was 99.26 ± 11.258, 30.13 ± 3.583 and 55.99 ± 7.074, respectively. The sub-dimensional statistical results are shown in Table 2, respectively.

**Table 2:** Results for the AWQ questionnaire, subscales, and subscale dimensions

Questionnaire	Dimensions	Mean	SD
AWQ total score (points)		185.37	20.034
AWQ subscales ^a^			
Work condition ^b^		99.26	11.258
	Leadership and unity	34.35	4.961
	Work environment	20.11	2.818
	Interpersonal relationship	12.39	1.987
	External environment	8.08	1.484
	Empowerment	10.48	2.414
	Social bond	8.77	1.084
	Workload	5.68	1.689
Work contents ^c^		30.13	3.583
	Working pace	13.30	1.607
	Familiarity	8.58	1.322
	Mental activity	8.25	1.364
Job satisfaction ^d^		55.99	7.074
	Consequence	28.27	4.254
	Approbation	21.48	2.669
	Status	6.24	1.719

^a^ AWQ has possible ranges of 46 to 230

^b^ AWQ work condition subscale has possible ranges of 25 to 125

^c^ AWQ work contents subscale has possible ranges of 7 to 35

^d^ AWQ job satisfaction subscale has possible ranges of 14 to 70

### Predictors of long-term care workers perceived professional attractiveness

Spearman correlation, Mann-Whitney U test, and Kruskal-Wallis H test were used to examine the professional attractiveness related to all variables and the results are shown in Table 3. In terms of demographics, the results showed that the differences in total professional attractiveness score by age, marital status, gender, education level, employment mode, years of work experience, nursing assistant certificate level, shift status, average daily care number, and average daily care disabled number were statistically significant (*p* < .05).

As for job institutional security and subjective perception factors, it was discovered that the more salary (*r* = .139, *p* = .006) and training opportunities (*r* = .226, *p* = .000) a long-term care worker gets and the higher perceived professional attractiveness, and the higher ageism in long-term care workers is associated with lower professional attractiveness (*r*=-.315, *p* = .000). However, there was no significant relationship between job burnout and professional attractiveness.

**Table 3:** Univariate analysis of demographics, job institutional security factors, subjective perception factors, and professional attractiveness

Variables	*N* (%)/M (IQR)	test	*P* value
Age	52(46, 56)	.244^c^	0.000*
Marital status			
Single	14(3.6%)	8.920^a^	0.030*
Married	344(87.8%)		
Divorced	3(1.0%)		
Widowed	31(8.0%)		
Gender			
Female	350(89.3%)	5589.500^b^	0.011*
Male	42(10.7%)		
Level of education of care worker			
Primary school and below	78(19.9%)	13.078^a^	0.011*
Junior high school	157(40.1%)		
High school/junior college	90(23.0%)		
Associate	46(11.7%)		
Bachelor’s degree and above	21(5.4%)		
Type of employment			
Contract worker	192(49.0%)	15668.500^b^	0.002*
Temporary worker	200(51.0%)		
Years of work	5(3, 7)	.353^c^	0.000*
Certificate level of care worker			
Certificateless	125(31.9%)	16.756^a^	0.002*
Junior elderly care worker	177(45.2%)		
Intermediate elderly care worker	63(16.1%)		
Senior elderly care worker	17(4.3%)		
Technician elderly care worker	10(2.6%)		
Shift system			
8-hour	95(24.2%)	26.560^a^	0.000*
12-hour	186(47.4%)		
24-hour	111(28.3%)		
Average daily number of caregivers	6(5, 10)	.216^c^	0.000*
Average number of persons with disabilities cared for daily	3(2, 5)	.262^c^	0.000*
Whether to participate in management			
Yes	118(30.1%)	15932.000^b^	0.820
No	274(69.9%)		
Monthly income (yuan)	3650(3025, 4000)	.139^c^	0.006*
Training times	5(3, 12)	.226^c^	0.000*
Ageism	58(51, 63)	− .315^c^	0.000*
Job burnout	50(45, 55)	.021^c^	0.679

^a^ Kruskal-Wallis H test

^b^ Mann-Whitney U test

^c^ Spearman correlation

Five significant variables in univariate analysis were entered into the regression equation and accounted for 26.9% of the variance in the scores of professional attractiveness.

As shown in Table 4, age(*β* = 0.129, *p*<.05), years of work experience(*β* = 0.156, *p*<.05), and 12-hour shifts(*β* = 0.185, *p*<.05) were found to have a positive relationship with long-term care workers perceived professional attractiveness. Training times per year(*β* = 0.148, *p*<.05), the only significant job institution security factor, was positively associated with the professional attractiveness. Whereas the ageism is higher for decreasing professional attractiveness total score(*β*=-0.267, *p*<.05).

**Table 4:** Predictors of long-term care workers perceived professional attractiveness

Independent variables	B	SE	β	t	*p*-value
Constant	188.534	11.129		16.940	0.000
Age	0.280	0.141	0.129	1.991	**0.047**
Years of work	0.749	0.242	0.156	3.095	**0.002**
Shift system(8-hour)					
12-hour 24-hour	7.420 -1.440	2.519 2.912	0.185 − 0.032	2.946 − 0.495	**0.003** 0.621
Training times	0.732	0.240	0.148	3.047	**0.002**
Ageism	− 0.620	0.110	− 0.267	-5.614	**0.000**

*Adjusted *R*^*2*^ = 0.269, *F* = 7.525

## Discussion

The present study has been the first to quantitatively describe the professional attractiveness among long-term care workers who work in NHs and explore the determinants from multiple aspects, such as job institutional security and subjective perception.

In this study, the culturally adapted AWQ was first used to evaluate the professional attractiveness of long-term care workers. Compared with the mean score of the whole questionnaire and each component, the findings suggested that the overall perceived professional attractiveness, as well as each component, were superior among long-term caregivers, which indicated that NH care workers find their profession as a decent attraction. This result was similar to previous study. Liu et al. employed a self-designed questionnaire on the attractiveness of the elderly care service talents and found that the attractiveness of elderly caregivers was generally acceptable [35]. Similarly, studies have shown that in the United States, low-income older workers report that working in long-term care is attractive. However, they preferred to work in a home environment compared to a nursing home or other institutional setting [36]. However, the results of another study suggest that care workers in LTC facilities do not rate their jobs highly due to low wages and intense job content [37]. Long-term care workers in Chinese NHs tend to be middle-aged and older women who have retired, been laid off, or migrated to cities to work, it is not easy to find a suitable job [38]. In the 1990s, many workers in state-owned factories were laid off in the course of market reforms and were left in a precarious situation due to the lack of re-employment programs. Many of these workers entered the care sector as a last resort due to the high demand and low barriers to employment in the care sector in urban areas [39]. They are satisfied with possessing a job, so that the LTC industry is relatively more desirable to them. Besides, the flexible shifts in NHs allow care workers relative freedom to arrange their own time, which might be sufficient for long-term care workers to have a better appreciation of their work.

Our study found that long-term care workers who are older and have longer working years perceived higher professional attractiveness. These findings are consistent with previous studies [40]. This may be attributable to the fact that care workers in NHs who are older and with longer work tenure already have a wealth of theoretical knowledge and practical solid ability to care for the elderly and are familiar with to work environment and content.

Besides, higher professional attractiveness is obtained when long-term care workers on 12-hour shifts compared to 8-hour shifts. Previous literature points out that when wards are on 12-hour nursing shifts, there is even decreased turnover intention in the LTC industry [41], which is similar to our findings. Whereas other studies indicated that longer shifts are associated with job burnout, dissatisfaction, and turnover intention [42]. This may endorse that too frequent shifts make it difficult for care workers in NHs to balance their work and life. Along with the disruption of circadian rhythms, short shifts in LTC facilities tend to prevent the formation of everyday routines and even lead to work-family conflict among care workers [10]. While working too many hours in a row easily fatigues employees and increases the risk of work errors, which could bring unpleasant experiences to care workers [43].

According to our study, long-term care workers who receive more training opportunities per year perceive greater attractiveness. Similarly, previous studies reported that the absence of training to enhance professionalism, as a characteristic of elderly care work, is associated with turnover [44]. The lack of professional training leaves care workers with less expertise in caring for the elder with or without disability or dementia, which puts them under tremendous pressure practically and psychologically. As a result, care workers may be dissatisfied with work condition nursing homes provided and even think about leaving their job, indicating that a career in LTC is no longer attractive to them [11]. Instead, training facilities capacity enhancement and increases accomplishment, which is an essential factor affecting work enthusiasm [45]. It has been suggested that satisfying the needs of knowledge acquisition of health professionals involved in the care of older adults is helpful in increasing work engagement and reducing job burnout [46]. The lack of professionalism and preparedness among care workers in long-term care sector reinforced the importance of training to create more promotion opportunities [40].

Additionally, our findings suggest that there is a decrease in professional attractiveness when care workers in NHs exhibit higher ageism. Ageism as a stereotype is prevalent in LTC facilities, and it may be linked to cognitive prejudice resulting from the low qualifications and dissatisfaction caused by low wages among LTC workers [47–49]. Age-based discrimination from care workers lead to decreased quality of care as well as psychological distress for the elderly [50]. LTC workers showing high levels of ageism tend to be reluctant to stay with and provide comprehensive care to older people, indicating that their jobs become less appealing and desirable [24].

### Limitations

This study has several limitations. Firstly, participants were selected only from two cities in China, which could introduce potential bias due to regional disparity and limit the generalizability of our results. Another important limitation is that the study did not consider the potential influence of varying characteristics of LTC facilities (e.g. business nature of LTC facilities), which could lead to incomplete results. Besides, the current study was conducted in the midst of the COVID-19 epidemic, and previous studies have demonstrated that changes in patterns of care brought about by COVID-19 epidemics can increase the workload of nursing home care workers [51]. In contrast, our findings cannot exclude the impact of the epidemic on the perceived professional attractiveness of nursing home care workers, which also implies that further investigations are necessary during non-epidemic periods. Additionally, it is impossible to establish causality due to the cross-sectional design.

### Implications

Measures such as developing professional education programs, lowering entry barriers, and providing monetary incentives have all been taken by the Chinese government in order to attract talent and solve the problem of labor shortage [52]. However, to maximize the attractiveness and retention of talent in the elderly care industry while improving the quality of care of LTC facilities, these measures are far from sufficient.​ By exploring the potential influences on the perceived professional attractiveness of long-term care workers, the current study provides a direction for policymakers and managers of long-term care institutions to consider improvements. Training programs, not only on nursing skills but also on knowledge of aging, should be prioritized to improve the ability of care workers to provide quality care and to mitigate ageism, potentially reversing unattractive career prospects. This will require greater government investment in education and training, as well as monitoring of the effective implementation of policies in LTC facilities. Additionally, managers of LTC institutions should consider a series of effective coping and managing mechanisms to attract talent. Career promotion systems and incentive mechanisms that go beyond the traditional ones should be put in place, with pay scales set according to the level of vocational skills qualification, years of experience, and working performance, to enhance the enthusiasm of elderly care workers. We have highlighted the importance of suitable working hours for ensuring care workers that are more likely to behave in high efficiency. Hence, administrators should optimize shift settings and try to avoid employees working too long hours in a row, maximizing job satisfaction of care workers while ensuring the quality of care, which in turn increases their perceived professional attractiveness.

Future research should include larger cross-regional samples and longitudinal studies to further verify relevant predictors and examine relationships across time. Besides, other LTC settings beyond nursing homes need to be considered, such as LTC hospitals. Additionally, future studies should explore professional attractiveness of long-term care workers from a qualitative perspective.

## Conclusions

This study examines the current state of professional attractiveness among LTC care workers in nursing homes. It also assessed the factors associated with professional attractiveness. This study provides insight into the plight faced by China’s LTC industry and strategies to improve the attractiveness of LTC industry to retain and appeal to healthcare professionals.

## Data Availability

The datasets analysed during the current study are available from the corresponding author on reasonable request.

## Abbreviations

NH: Nursing Home
LCT: Long-term Care
FSA: Fraboni Scale of Ageism
AWQ: The Attractive Work Questionnaire
MBI: The Maslach Burnout Inventory

## References

[1] United Nations, Department of Economic and Social Affairs, Population Division. (2019): World Population Prospects 2019: Highlights (ST/ESA/SER.A/423).; 2019.

[2] National Bureau of Statistics of China. Communiqué of the Seventh National Population Census (No. 5). http://www.stats.gov.cn/sj/tjgb/rkpcgb/qgrkpcgb/202302/t20230206_1902005.html. Accessed 6 May 2023.

[3] Ge Y, Wang L, Feng W, Zhang B, Liu S, Ke Y (2020). The challenge and strategy selection of healthy aging in China. J Manage World.

[4] Perracini MR, Arias-Casais N, Thiyagarajan JA, Rapson C, Isaac V, Ullah S, Hyobum J, Sadana R, Han ZA (2022). A recommended Package of Long-Term Care services to promote healthy ageing based on a WHO Global Expert Consensus Study. J AM MED DIR ASSOC.

[5] Wan H, Wang Y, Fang L, Tao L, Li S, Yang Y, Yang C (2018). Study on integrated health care under institution-community-home endowment mode. Chin Health Resour.

[6] Chen L, Zhang X, Xu X (2020). Health Insurance and Long-Term Care Services for the disabled Elderly in China: based on CHARLS Data. RISK MANAG HEALTHC P.

[7] Fang EF, Xie C, Schenkel JA, Wu C, Long Q, Cui H, Aman Y, Frank J, Liao J, Zou H et al. A research agenda for ageing in China in the 21st century (2nd edition): Focusing on basic and translational research, long-term care, policy and social networks. *AGEING RES REV* 2020, 64:101174. 10.1016/j.arr.2020.101174 PMC7505078 32971255

[8] Wang L (2014). A study on the Development of China’s Nursing Home Services in Urban Area. Popul J.

[9] Two Departments Promulgate and Implement the National Occupational Skills Standard for Elderly Caregivers. (2019 Edition). https://www.gov.cn/xinwen/2019-10/17/content_5440977.htm. Accessed 8 April 2024.

[10] Hauser C, Stahl J, Simon M, Valenta S, Favez L, Zuniga F. Identifying work-related factors associated with work-family conflict of care workers in nursing homes: a cross-sectional study. J ADV NURS 2023. 10.1111/jan.15704 37209293

[11] Pardo-Garcia I, Martinez-Lacoba R, Escribano-Sotos F. Socioeconomic factors related to job satisfaction among Formal Care Workers in nursing homes for older dependent adults. INT J ENV RES PUB HE 2021, 18(4). 10.3390/ijerph18042152 PMC7927119 33672101

[12] Zeng Y, Hu X, Li Y, Zhen X, Gu Y, Sun X, Dong H. The quality of caregivers for the Elderly in Long-Term Care Institutions in Zhejiang Province, China. INT J ENV RES PUB HE 2019, 16(12). 10.3390/ijerph16122164 PMC6617400 31248074

[13] Dai F, Zhang X, Wan Q (2014). Willingness and influencing factors for senior nurses involving in long-term care for the elderly. Chin Gen Pract.

[14] Darling R, Sendir M, Atav S, Buyukyilmaz F (2018). Undergraduate nursing students and the elderly: an assessment of attitudes in a Turkish university. GERONTOL GERIATR EDU.

[15] Rust TB, Wagner LM, Hoffman C, Rowe M, Neumann I (2008). Broadening the patient safety agenda to include safety in long-term care. Healthc Q.

[16] Ateg M, Andersson I, Rosen G (2009). Change processes for attractive work in Small Manufacturing companies. Hum FACTORS Ergon Manuf.

[17] Qi X, Wang Q, Zhao H, Kong L, Fan J, Li J (2022). A conceptual analysis of long-term caregivers’ occupational attractiveness. Chin J Mod Nurs.

[18] Kash BA, Naufal GS, Dagher RK, Johnson CE (2010). Individual factors associated with intentions to leave among directors of nursing in nursing homes. HEALTH CARE MANAGE R.

[19] Aloisio LD, Coughlin M, Squires JE (2021). Individual and organizational factors of nurses’ job satisfaction in long-term care: a systematic review. INT J NURS STUD.

[20] Choi J, Flynn L, Aiken LH (2012). Nursing practice environment and registered nurses’ job satisfaction in nursing homes. GERONTOLOGIST.

[21] Foa C, Guarnieri MC, Bastoni G, Benini B, Giunti OM, Mazzotti M, Rossi C, Savoia A, Sarli L, Artioli G (2020). Job satisfaction, work engagement and stress/burnout of elderly care staff: a qualitative research. Acta Biomed.

[22] Lee S, Oh G, Fabius CD, Reckrey JM (2023). Working conditions Affecting Home Care workers’ stress and turnover intention. J APPL GERONTOL.

[23] Butler RN (1969). Age-ism: another form of bigotry. GERONTOLOGIST.

[24] Liu CC, Liu LF, Chuang SS (2022). The Effect of Ageist behaviors on Home Care workers’ job satisfaction and Retention in Long-Term Care. J APPL GERONTOL.

[25] Campbell SL (2010). The attractiveness of a career in geriatric nursing. J GERONTOL NURS.

[26] White EM, Aiken LH, McHugh MD (2019). Registered nurse burnout, Job Dissatisfaction, and missed care in nursing homes. J AM GERIATR SOC.

[27] Ohara Y, Nomura Y, Yamamoto Y, Okada A, Hosoya N, Hanada N, Hirano H, Takei N. Job Attractiveness and Job Satisfaction of Dental Hygienists: From Japanese Dental Hygienists’ Survey 2019. INT J ENV RES PUB HE 2021, 18(2). 10.3390/ijerph18020755 PMC7830899 33477353

[28] Bjorn C, Josephson M, Wadensten B, Rissen D (2015). Prominent attractive qualities of nurses’ work in operating room departments: a questionnaire study. WORK.

[29] Tabachnick BG, Fidell LS. *Using multivariate statistics* |.*6**6*. 7th edition. Boston: MA: pearson; 2013.

[30] Fraboni M, Saltstone R, Hughes S (1990). The Fraboni Scale of Ageism (FSA): an attempt at a more precise measure of Ageism. Can J Aging / La Revue Canadienne Du Vieillissement.

[31] Fan J, Zhao H, Liu Y, Kong L, Mao J, Li J (2020). Psychometric properties of a Chinese version of the Fraboni scale of ageism: evidence from medical students sample. BMC MED EDUC.

[32] Maslach C, Schaufeli WB, Leiter MP (2001). Job burnout. ANNU REV PSYCHOL.

[33] Li C, Shi K. The impact of Distributive and Procedural Equity on Job Burnout. ACTA PSYCHOL SIN 2003(05):677–84.

[34] ÅTEG M, HEDLUND A. *Researching attractive work. Analyzing a model of attractive work using theories on applicant attraction, retention and commitment*. 1th edition: Linnéuniversitet: Repro Copycenter; 2010.

[35] Liu Z, Zhang Y (2016). A Preliminary Investigation and Analysis on the attractiveness of the Pension Service Industry to talents in China. Sci Res Aging.

[36] Hwalek M, Straub V, Kosniewski K (2008). Older workers: an opportunity to expand the long-term care/direct care labor force. GERONTOLOGIST.

[37] Kim B, Liu L, Ishikawa H, Park S (2019). Relationships between social support, job autonomy, job satisfaction, and burnout among care workers in long-term care facilities in Hawaii. EDUC GERONTOL.

[38] Yan Z (2020). An ethical glimpse into nursing Home Care Work in China: Mei Banfa. ETHICS SOC WELF.

[39] Yan Z (2022). I tried to control my emotions : Nursing Home Care Workers ' experiences of Emotional Labor in China. J CROSS-CULT GERONTO.

[40] Chenoweth L, Jeon YH, Merlyn T, Brodaty H (2010). A systematic review of what factors attract and retain nurses in aged and dementia care. J CLIN NURS.

[41] Li CC, Yamamoto-Mitani N (2021). Ward-level nurse turnover and related workplace factors in long-term care hospitals: a cross-sectional survey. J NURS MANAGE.

[42] Halter M, Boiko O, Pelone F, Beighton C, Harris R, Gale J, Gourlay S, Drennan V (2017). The determinants and consequences of adult nursing staff turnover: a systematic review of systematic reviews. BMC HEALTH SERV RES.

[43] Yang L, Tang C, Zhang W, Feng M, Wei P, Guo F, Gao Z (2020). Research on the correlation between clinicians’ working hours and medical errors. Chin Health Service Manage.

[44] Lim J. Characteristics of Elderly Care Work That Influence Care Workers’ Turnover Intentions. HEALTHCARE-BASEL 2021, 9(3). 10.3390/healthcare9030259 PMC7999930 33804491

[45] Colindres CV, Bryce E, Coral-Rosero P, Ramos-Soto RM, Bonilla F, Yassi A (2018). Effect of effort-reward imbalance and burnout on infection control among Ecuadorian nurses. INT NURS REV.

[46] Kubicek B, Korunka C, Ulferts H (2013). Acceleration in the care of older adults: new demands as predictors of employee burnout and engagement. J ADV NURS.

[47] North MS, Fiske ST (2012). An inconvenienced youth? Ageism and its potential intergenerational roots. PSYCHOL BULL.

[48] Reyna C, Goodwin EJ, Ferrari JR (2007). Older adult stereotypes among care providers in residential care facilities: examining the relationship between contact, eduaction, and ageism. J GERONTOL NURS.

[49] Sao JJ, Amado CA (2017). On studying ageism in long-term care: a systematic review of the literature. INT PSYCHOGERIATR.

[50] Xu D, Wang Y, Li M, Zhao M, Yang Z, Wang K. Depressive symptoms and ageism among nursing home residents: the role of Social Support. INT J ENV RES PUB HE 2022, 19(19). 10.3390/ijerph191912105 PMC9564776 36231405

[51] Lai VS, Yau SY, Lee LY, Li BS, Law SS, Huang S. Caring for Older People during and beyond the COVID-19 Pandemic: Experiences of Residential Health Care Workers. *INT J ENV RES PUB HE* 2022, 19(22). 10.3390/ijerph192215287 PMC9692584 36430006

[52] Feng Z, Glinskaya E, Chen H, Gong S, Qiu Y, Xu J, Yip W (2020). Long-term care system for older adults in China: policy landscape, challenges, and future prospects. Lancet.

